# The role of miR-152 in urological tumors: potential biomarkers and therapeutic targets

**DOI:** 10.3389/fimmu.2024.1464327

**Published:** 2024-11-13

**Authors:** Xin Li, Biao Qian, Xu Chen, Maolei Shen, Shankun Zhao, Xinsheng Zhang, Jian He

**Affiliations:** ^1^ Department of Urology, Taizhou Central Hospital (Taizhou University Hospital), Taizhou, Zhejiang, China; ^2^ Department of Urology, First Affiliated Hospital of Gannan Medical University, Ganzhou, Jiangxi, China; ^3^ Department of Pathology, The Third Affiliated Hospital of Zhengzhou University, Zhengzhou, Henan, China

**Keywords:** miR-152, urological malignancy, therapeutic target, biomarker, microRNA

## Abstract

Urological malignant tumors pose a significant threat to human health, with a high incidence rate each year. Prostate cancer, bladder cancer, and renal cell carcinoma are among the most prevalent and extensively researched urological malignancies. Despite advancements in research, the prognosis for these tumors remains unfavorable due to late detection, postoperative recurrence, and treatment resistance. A thorough investigation into their pathogenesis is crucial for early diagnosis and treatment. Recent studies have highlighted the close association between microRNAs (miRNAs) and cancer progression. miRNAs are small non-coding RNAs composed of 19-23 nucleotides that regulate gene expression by binding to the 3’ untranslated region (3’UTR) of target mRNAs, impacting key cellular processes such as proliferation, differentiation, apoptosis, and migration. Dysregulation of miRNAs can disrupt the expression of oncogenes and tumor suppressor genes, contributing to cancer development. Among the various miRNAs studied, miR-152 has garnered attention for its role in urological malignancies. Several studies have indicated that dysregulation of miR-152 expression is significant in these cancers, warranting a comprehensive review of the evidence. This review focuses on the expression and function of miR-152 in prostate cancer, bladder cancer, and renal cell carcinoma, elucidating its mechanisms in cancer progression and exploring its potential as a therapeutic target and biomarker in urological malignancies.

## Introduction

Urological malignancies encompass prostate cancer, bladder cancer, renal cell carcinoma, penile cancer, testicular cancer, and uroepithelial cancer. According to GLOBOCAN, there were approximately 2.5 million new cases of urological malignancies globally in 2022, representing 12.6% of all cancers. These cases resulted in 770,000 deaths, accounting for 8% of all cancer-related deaths (1). The number of new cases and deaths of urinary malignant tumors in 2022 has exceeded the number published by GLOBOCAN in 2020 (2). Prostate cancer, bladder cancer, and renal cell carcinoma are among the most prevalent and extensively researched urological malignancies. Prostate cancer, the second most common cancer in men, was responsible for 1,466,680 new cases and 396,792 deaths in 2022. The majority of patients with prostate cancer progress to desmoplasia-resistant prostate cancer, leading to a poor prognosis. Bladder cancer, the second most common urological malignancy, ranked ninth in terms of incidence in 2022 (1) Renal cell carcinoma, although less common than prostate and bladder cancer, saw 434,419 new cases and 155,702 deaths in 2022, ranking 14th among all cancers (1). The incidence of urological malignancies is expected to rise globally due to population growth and aging (3). These malignancies pose a significant burden on global health and finances, with the United States estimated to have spent $314.7 billion in 2020 on the prevention and treatment of urological malignancies (4).

Early urological malignancies are often treated with surgical resection, which boasts a high success rate. However, the lack of specific symptoms in the early stages leads to most patients being diagnosed at an advanced stage. Additionally, some non-invasive biomarkers, like prostate specific antigen (PSA), have limitations in the early diagnosis of prostate cancer, such as low specificity and overdiagnosis (5, 6). Urine cytological testing is even less sensitive for diagnosing bladder cancer, while cystoscopic biopsy is invasive and inconvenient (7). This highlights the urgent need for sensitive diagnostic methods to aid in early urological tumour diagnosis. For patients with advanced cancer, chemotherapy and radiotherapy are commonly used to slow disease progression and improve quality of life. Unfortunately, cancer often develops resistance to these treatments after several courses, posing a significant challenge (8). Despite recent advancements in immunotherapy and targeted therapies, challenges persist due to drug-resistant gene mutations post-targeted therapies, immune adverse effects post-immunotherapy, and limited use of targeted drugs due to high costs. Thus, there is a pressing need to develop new therapeutic strategies for urological tumours, necessitating a comprehensive understanding of their pathogenesis.

In the past two decades, the study of miRNAs has sparked a molecular revolution, with numerous studies demonstrating their significant role in cancer. miRNAs impact essential cellular processes like cell proliferation, differentiation, cell cycle regulation, invasion, metastasis, and angiogenesis by modulating the expression of target genes. Given the close connection between these processes and cancer, miRNAs are intricately linked to cancer development. Among the well-researched miRNAs in oncology, miR-152 stands out for its consistent oncogenic role across various cancer types, including colorectal (9, 10), gastric (11, 12), hepatocellular (13, 14), lung (15–17), breast (18, 19), ovarian (20, 21), cervical (22, 23), and glioma (24, 25). While studies on the relationship between miR-152 and urological tumors abound, a comprehensive review on this subject is lacking. This paper aims to fill this gap by examining the impact of miR-152 on biological behaviors of cancer cells in urological tumors, such as proliferation, invasion, metastasis, angiogenesis, and apoptosis. It also explores the potential of miR-152 as a biomarker for early diagnosis, treatment, and prognosis of urological tumors, as well as its therapeutic implications in miRNA-based cancer treatments. By delving into the role of miR-152, this review seeks to enhance our understanding of the pathogenesis and therapeutic strategies for urological malignancies.

## Overview of miRNAs

**Figure 1:**
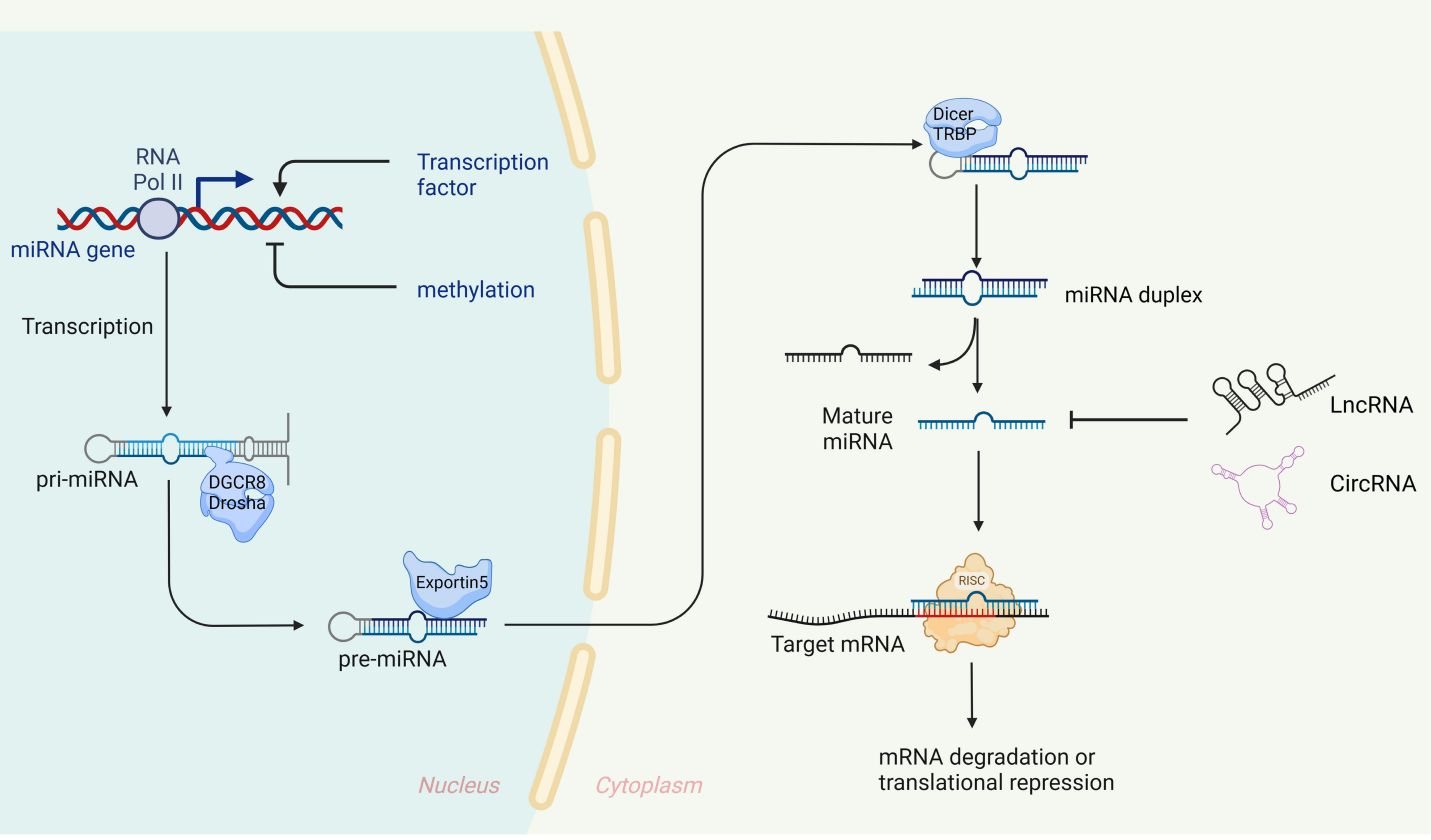
Biogenesis and expression regulation of miRNAs. In the nucleus, primary miRNA (pri-miRNA), which possesses specific structural characteristics, is transcribed by RNA polymerase II. These pri-miRNAs are then cleaved by a microprocessor complex, composed of Drosha and its cofactor DGCR8, to generate precursor miRNAs (pre-miRNAs) of a defined length (120–123), The pre-miRNAs are transported to the cytoplasm via Exportin-5 (124, 125), where Dicer and TRBP further process them into double-stranded RNA molecules (126, 127). These molecules then unwind to form mature single-stranded miRNAs, with one strand designated as ‘5p’ and the other as ‘3p’. Finally, these mature miRNAs are integrated into the RNA-induced silencing complex (RISC) with the assistance of argonaut 2 protein (AGO2) (128). miRNAs with nucleotide sequences 2-7, known as ‘seed’ sequences, are directed by AGO2 to recognize and bind the 3’UTR of target mRNA (129), When fully complementary, the target mRNA is degraded (130), when not fully complementary, translation is inhibited (32).

miRNAs were initially discovered in 1993 in the nematode Caenorhabditis elegans (26), Since then, a growing number of miRNAs have been identified and characterized, with over 28,000 miRNAs currently known across a variety of organisms (27). These small RNAs, approximately 19-23 nucleotides in length, are evolutionarily conserved and play a crucial role in gene expression regulation. They typically bind to the 3’ untranslated region (3’ UTR) of target mRNA, leading to either degradation or inhibition of translation (28, 29). In some rare instances, miRNAs can enhance translation of target genes, contributing to post-transcriptional gene regulation (3, 30, 31). It is estimated that miRNAs regulate at least one-third of all genes (32). The biogenesis of miRNA is an extremely complex process, as shown in **Figure 1**.

miRNAs play a crucial role in regulating cellular processes, with their dysregulation linked to various diseases, including cancer. The connection between cancer and dysregulated miRNA expression was initially observed in patients with chronic lymphocytic leukaemia (CML), where the absence of chromosomal regions coding for miR15 and miR16 was noted (33). Cancer-associated miRNAs can be broadly categorized into oncogenic miRNAs (oncomiRs) and tumor suppressor miRNAs (miRsupps) (34, 35). OncomiRs promote cancer progression by influencing cell proliferation, apoptosis, and other cancer-related processes, typically exhibiting overexpression in cancers (36). Conversely, miRsupps impede malignant behaviors in cancer cells, thus hindering cancer progression, and are usually underexpressed in cancer (37). The roles of specific miRNAs can vary across different cancer types. For instance, miR-155 was found to be overexpressed in renal cell carcinoma, promoting proliferation and metastasis (38), while in malignant melanoma, its overexpression inhibited cancer progression (39). In addition, miR-137 functions as a tumor suppressor in various cancers, including lung cancer (40), gastric cancer (41), ovarian cancer (42), endometrial cancer (42), and nervous system tumors (43). However, some studies have indicated that miR-137 can also act as an oncomiR, promoting cancer progression (44, 45). Notably, the same miRNA may exhibit contradictory roles within the same cancer, potentially influenced by factors such as technological variations, diverse targets, and cancer subtypes.

## Function and expression regulation of miR-152

miR-152, a member of the miR-148/152 family along with miR-148a and miR-148b, was initially discovered in the mouse colon through tissue-specific cloning (46), These three members share the same seed sequence, leading to the targeting of genes with immediate homology. Variations in reported miRNA targets across studies could be attributed to the focus on individual miRNAs within the family without considering others (47). Moreover, the 3’-terminal non-seeded sequence of miRNAs plays a role in stabilizing their binding to target genes (48), suggesting that the miR-148/152 family has the potential to target different mRNAs based on their distinct non-seeded sequences.

Aberrant expression of miR-152 has been observed in various oncological and non-oncological diseases. For instance, Nielsen et al. identified 12 upregulated human miRNAs, including miR-152, in the serum of patients with type 1 diabetes (49). Moreover, increased miR-152 expression has been detected in atheromatous plaque tissue and classical monocytes (50). Research has shown that miR-152 overexpression promotes lipid accumulation in preadipocytes, correlating with *in vivo* adipogenesis and intramuscular adipogenesis, indicating its potential relevance in obesity and obesity-related metabolic syndrome (51). Furthermore, up-regulation of miR-152-5p expression was noted in the mandible of a rat osteoporosis model, suggesting its involvement in regulating osteogenic differentiation by targeting the autophagy-related protein ATG14. These findings collectively demonstrate the diverse functions of miR-152 and contribute to our understanding of its significance in non-tumour diseases.

miR-152 has been extensively studied in various types of tumors, showing reduced expression and potential as a tumor suppressor miRNA. For instance, in hepatocellular carcinoma, miR-152-3p was significantly down-regulated in tumor tissues compared to non-tumor tissues. Overexpression of miR-152-3p inhibited ROBO1, blocking the malignant characteristics of hepatocellular carcinoma (14). Similarly, in gastric cancer, reduced expression of miR-152-3p led to inhibited proliferation, clone formation, migration, and induced apoptosis in cancer cells (52). In cholangiocarcinoma, miR-152 targeted DNMT1, with reduced expression in cancer cells (53). Notably, miR-152 was also found to be down-regulated in ovarian cancer and correlated with the malignant phenotype of ovarian cancer cells (54). Reduced miR-152 expression was associated with poorer survival outcomes in endometrial plasma adenocarcinoma (55). Furthermore, in lung cancer miR-152 targets TNS1 to regulate the Akt/mTOR/RhoA pathway, inhibiting the progression of non-small cell lung cancer (15). In breast cancer, miR-152 inhibits cell survival and promotes apoptosis by targeting EPAS1, enhancing the sensitivity of breast cancer cells to paclitaxel (56). Kong et al. found that miR-152 inhibits glioma progression and tumorigenesis by targeting FBXL7, and increases the cytotoxicity of temozolomide-induced glioma cells (24). Overall, miR-152 shows decreased expression in digestive system tumors, reproductive system tumors, lung cancer, breast cancer, and glioma, with this decreased expression correlating with the malignant phenotype of cancer cells. Restoring miR-152 expression inhibits cancer progression and increases the sensitivity of cancer cells to chemotherapeutic agents, indicating its role as a tumor-suppressor miRNA in various cancers. Therefore, miR-152 may serve as a potential target in cancer therapy.

The expression and activity of miR-152 are tightly regulated both temporally and spatially in normal physiological conditions. Disruption of this regulation is closely linked to various human diseases, such as cancer growth and metastasis (57). Several mechanisms can lead to dysregulation of miRNAs, including epigenetic changes, abnormal binding of transcription factors, interference with miRNA production, post-transcriptional modifications of RNA, and RNA degradation (37, 58). There is growing evidence of epigenetic connections between DNA methylation changes and miR-152 levels in cancer. miR-152 is often silenced in different types of cancer, like prostate (59), bladder (60), colorectal (61), breast (62) endometrial (63), and glioblastoma (25), due to DNA hypermethylation. This hypermethylation is driven by the increased activity of DNMT1, an enzyme responsible for maintaining DNA methylation levels. Interestingly, DNMT1 is a target of miR-152, and its levels are inversely related to miR-152 in many cancers (64, 65), suggesting a negative feedback loop between them. miR-152 expression is also influenced by various transcription factors, such as ELF1, which can enhance miR-152-3p levels by directly interacting with its promoter (17). In breast cancer, the activation of miR-152 expression is triggered by the translocation of the β-linker protein and PKM2 complex into the nucleus in response to IGF-1 (66). Moreover, the activity of miR-152 is regulated by competing endogenous RNAs (ceRNAs) that bind to miR-152 and reduce its inhibitory effects on downstream targets, primarily including certain lncRNAs, circRNAs, and pseudogene transcripts (67, 68).

## miR-152 in prostate cancer

**Figure 2:**
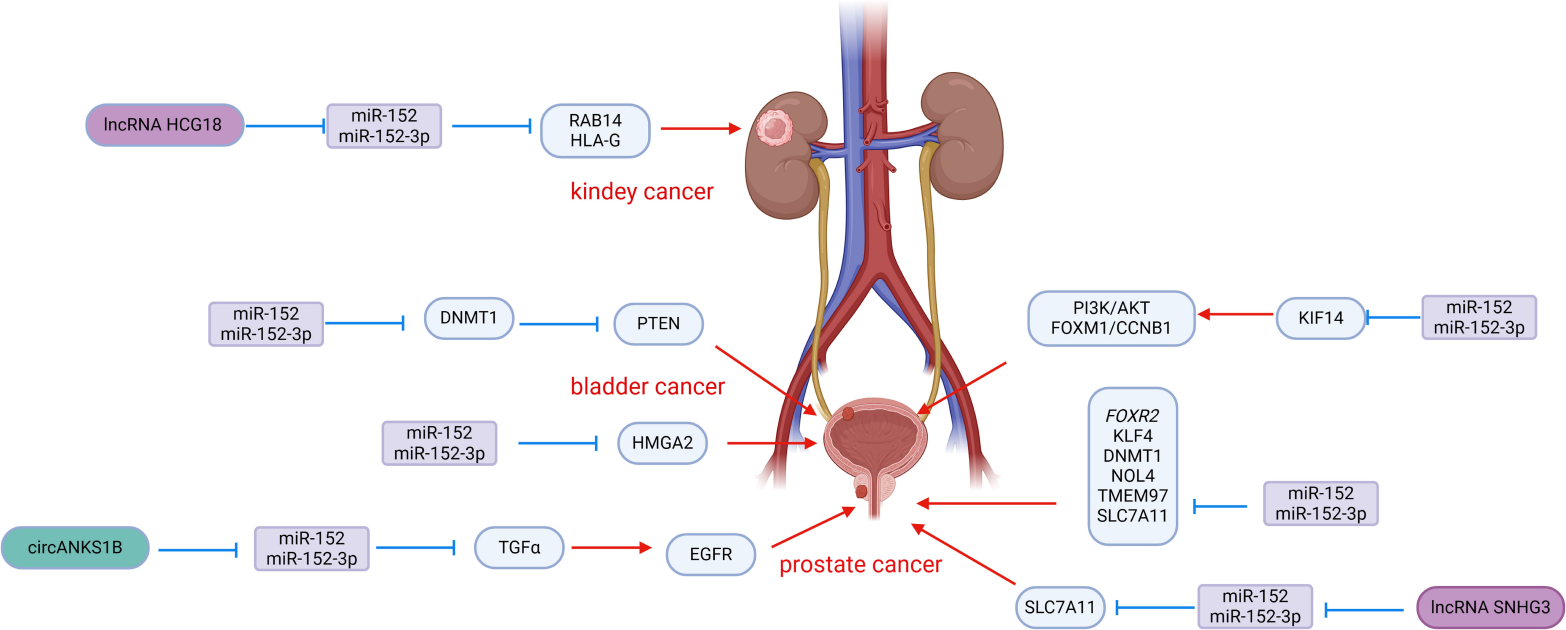
Roles of miR-152 and its targeted proteins and signaling cascades in the development of urologic malignancies.

Among the studies focusing on miR-152 and urological tumors, prostate cancer has been the most extensively researched. Numerous studies have demonstrated that miR-152 is downregulated in prostate cancer tissues and cell lines, and its altered expression is intricately linked to the development of prostate cancer (**Figure 2**). Restoring miR-152 expression has been shown to impede the progression of prostate cancer by targeting and suppressing genes and signaling pathways that are crucial in cancer development. This inhibition ultimately hinders the advancement of prostate cancer. The subsequent discussion provides a detailed explanation of the role and mechanism of miR-152 in prostate cancer.

Prostate cancer is known for its aggressive nature, with a strong invasive ability that allows it to infiltrate and metastasize to surrounding tissues, contributing to its poor prognosis and high rate of postoperative recurrence. Chen et al. conducted a study on miR-152 expression in 48 primary prostate cancers, finding a significant down-regulation compared to non-malignant control tissues. Specifically, patients with a Gleason score >7 showed lower levels of miR-152, which was also associated with the pathological stage of prostate cancer. Overexpression of miR-152 in PC-3 and DU145 cells effectively suppressed the invasive and migratory abilities of prostate cancer cells by targeting TGFα, a key player in EGFR signaling (69). The activation of EGFR is crucial for cell proliferation, angiogenesis, motility, and metastasis (70, 71), with previous research linking EGFR to invasion and metastasis in various tumors (72, 73). By inhibiting TGFα-EGFR signaling, miR-152 also downregulated downstream genes MMP2 and MMP9, further suppressing the invasive and migratory potential of prostate cancer cells *in vitro* (69). Interestingly, the tumor-suppressive effects of miR-152 could be counteracted by circANKS1B, which acts as a sponge for miR-152, binding to miR-152-3p and attenuating the inhibition of TGFα. This mechanism promotes the invasion and metastasis of prostate cancer cells, highlighting the intricate interplay between miR-152, TGFα, and circANKS1B in prostate cancer progression (74). Xu et al. reported that miR-152 targets the 3′UTR of FOXR2 in prostate cancer, leading to downregulation of FOXR2 expression, which in turn inhibits cell proliferation and induces apoptosis. Additionally, lncRNA HOTAIR was observed to interact with miR-152, resulting in upregulation of FOXR2 and further promoting prostate cancer progression (75).

Ramalho-Carvalho et al. employed a combined approach involving micro-RNA expression analysis and differential methylation localization to identify novel miRNAs downregulated by aberrant DNA methylation in prostate cancer. They discovered a transcription unit comprising COPZ2-miR-152-3p (76). Their study highlighted that the downregulation of miR-152-3p is a common characteristic of prostate cancer and plays a role in sustaining malignant traits and tumor growth. Functional assays conducted *in vitro* revealed that overexpression of miR-152-3p led to a significant decrease in cell viability in LNCaP and PC3 cells, along with an increase in cell accumulation in S and G2/M phases. Moreover, the overexpression of miR-152-3p resulted in the downregulation of several cell cycle regulators at the transcriptional level. Additionally, miR-152-3p overexpression suppressed the expression of epithelial-mesenchymal transition-related genes TWIST and VIM, leading to a notable reduction in the invasive capacity of PC3 cells. Conversely, miR-152-3p mimics induced apoptosis in both cell lines (76). Mechanistically, NOL4 (nucleolin 4) and TMEM97 (transmembrane protein 97) were identified as targets of miR-152-3p. TMEM97 has been found to be up-regulated in various malignant tumors, including prostate cancer (77, 78). miR-152-3p also down-regulates the expression of genes involved in the MAPK/ERK, TFG-Beta, JAK-STAT3, and EMT pathways, which are classical pathways in cancer.

Feng et al. discovered a synergistic effect of miR-148-3p and miR-152-3p in prostate cancer. Their study revealed a reduction in the expression of both miR-148-3p and miR-152-3p in prostate cancer cells. Functional assays conducted *in vitro* showed that the combined overexpression of miR-148-3p and miR-152-3p had a greater inhibitory impact on cell proliferation and apoptosis induction compared to individual overexpression. This suggests a synergistic relationship between miR-148-3p and miR-152-3p in inhibiting the growth of PC3 and LNCaP cells (79). Mechanistically, miR-148-3p and miR-152-3p, being part of the same family, share a common seed sequence and target the 3′ UTR of KLF4. By jointly inhibiting KLF4, miR-148-3p and miR-152-3p effectively impede the progression of prostate cancer. This synergistic effect was further validated in prostate cancer xenograft mice (79).

miR-152 is present in prostate cancer and is involved in a feedback pathway with DNMT1, serving as a target gene for miR-152. miR-152 functions by repressing DNMT1 expression post-transcriptionally. Conversely, increased expression of DNMT1 results in widespread hypermethylation of DNA, leading to a notable decrease in miR-152 expression (47). Theodore et al. observed hypermethylation of the promoter region in the LNCaP and PC-3 cell lines, resulting in the inactivation of miR-152 through hypermethylation in prostate cancer. They proposed that miR-152, which acts as a tumor suppressor, is inactivated by methylation, indicating that the epigenetic regulation of miR-152/DNMT1 may have a significant impact on the aggressiveness of prostate cancer (59). Similarly, Gurbuz et al. found a substantial increase in DNMT1 gene expression levels and a notable decrease in DNMT3b and PTEN expression in patients with prostate cancer and metastatic prostate cancer. Linear regression analysis revealed a significant relationship between miR-152 and DNMT1/DNMT3b/PTEN, although this study did not confirm a direct association of miR-152 with these genes (80).

Wang et al. found that lncRNA SNHG3 expression was markedly upregulated in prostate cancer tissues and cell lines, correlating with poor prognosis. Functional assays revealed that SNHG3 overexpression promoted proliferation, migration, and invasion of prostate cancer cells while inhibiting apoptosis (81). Mechanistically, SNHG3 acts as a molecular sponge for miR-152-3p, competitively modulating the inhibition of miR-152-3p on its target SLC7A11. The upregulation of SLC7A11 reduces methionine-dependence in cancer cells, a common feature among cancer cells (82, 83). Methionine dependence refers to the inability of cells to grow in media containing homocysteine instead of methionine, suggesting methionine restriction as a potential cancer treatment strategy (84, 85). This study highlights the role of the SNHG3/miR-152-3p/SLC7A11 axis in promoting prostate cancer progression by influencing methionine-dependence, underscoring the significance of miR-152-3p in inhibiting prostate cancer (81).

In summary, miR-152 is frequently downregulated in prostate cancer tissues and cell lines. Acting as a tumor suppressor miRNA, miR-152 targets and suppresses pro-oncogenic factors, leading to the inhibition of prostate cancer cell growth, movement, and invasion. It also triggers cell cycle arrest and apoptosis, ultimately impeding the progression and spread of prostate cancer. These findings indicate that miR-152 holds promise as a potential target for prostate cancer therapy.

## miR-152 in bladder cancer

Bladder cancer is a prevalent malignancy among urological tumors, with approximately 70% of patients being diagnosed with non-muscle invasive bladder cancer. Out of these cases, 50%-70% face recurrence, and 10%-20% progress to muscle invasive bladder cancer with a poor prognosis (86) Despite recent advancements in understanding the mechanisms, diagnosis, and treatment of bladder cancer, the high recurrence rate and low survival rate of patients with muscle-invasive bladder cancer remain unresolved issues. Various studies have highlighted the significance of miR-152 in bladder cancer (**Figure 2**). Like in prostate cancer, miR-152 is often under-expressed in bladder cancer tissues and cell lines, functioning as a tumor suppressor miRNA. Zhang et al. observed significantly lower levels of miR-152 expression in bladder cancer cell lines T24 cells and UM-UC-3 cells compared to the normal urinary tract epithelial cell line SV-HUC-1. Moreover, miR-152 expression was notably lower in bladder cancer tissues compared to adjacent non-cancerous tissues. The reduced levels of miR-152 expression were found to be associated with the stage and grade of bladder cancer, and the overexpression of miR-152 effectively inhibited the growth of bladder cancer cells. The methylation rate of miR-152 promoter CpG islands was notably higher in bladder cancer cell lines and tissues compared to normal tissues. Mechanistic studies indicated that DNMT1-mediated hypermethylation was responsible for the decreased expression of miR-152 (60). Furthermore, DNMT1 was identified as a potential target of miR-152, with luciferase reporter gene assays showing direct binding of miR-152 to DNMT1 3′UTR, leading to its inhibition in bladder cancer cells (60). These findings suggest a reciprocal regulatory relationship between DNMT1 and miR-152 in bladder cancer, where increased DNMT1 levels cause hypermethylation of miR-152, resulting in its downregulation. Simultaneously, miR-152 suppresses DNMT1 expression by targeting its 3′-UTR. Additionally, research by Liu et al. revealed that DNMT1 promoted the promoter methylation of phosphatase and tensin homologue (PTEN), inhibiting PTEN expression. Knockdown of PTEN mitigated DNMT1’s effects on bladder cancer cell proliferation and migration, indicating that miR-152-3p inhibits DNMT1 expression, which in turn affects PTEN via DNA methylation regulation. The miR-152-3p/DNMT1/PTEN pathway plays a crucial role in bladder cancer development (65). These studies enhance our understanding of the intricate relationship between miR-152 and DNMT1 in bladder cancer. Kinesin family member 14 (KIF14) plays a crucial role in various biological processes. Studies have shown that overexpression of KIF14 is linked to the advancement and spread of different types of cancers (87, 88). Fang et al. reported that the overexpression of miR-152-3p and the knockdown of KIF14 inhibited the proliferation, migration, and invasion of bladder cancer cells while inducing apoptosis (89). Mechanistically, miR-152-3p directly targets KIF14, leading to a reduction in its expression, which in turn inhibits the PI3K/AKT and FOXM1/CCNB1 pathways, thereby impeding the progression of bladder cancer. The PI3K/AKT and FOXM1/CCNB1 pathways are well-established pathways involved in cancer development and cell cycle regulation (89). Moreover, miR-152-3p was found to target high mobility histone A2 (HMGA2), further inhibiting bladder cancer cell proliferation and invasion by suppressing HMGA2 expression (90). Additionally, Zhang et al. reported that lncRNA CCAT1 promotes the proliferation, migration, and invasion of bladder cancer cells. Further examination of its potential pathways revealed that miR-152-3p is associated with these functions. lncRNA CCAT1 may function as a sponge for miR-152-3p by binding to it, thereby weakening the activity of miR-152-3p and inhibiting its target genes, which in turn promotes the progression of bladder cancer (91).

## miR-152 in renal cell carcinoma

Renal cell carcinoma (RCC) is the third most common urological tumor with a high mortality rate. Clear renal cell carcinoma (ccRCC) is a predominant subtype, constituting approximately 80% of RCC cases (2). Compared with prostate cancer, research on miR-152 in renal cell carcinoma is limited. Several studies have highlighted the significance of miR-152 in RCC (**Figure 2**). Yang et al. reported that LncRNA HCG18 is highly expressed in clear cell renal cell carcinoma (ccRCC) cells and tissues. Further research identified miR-152-3p as a downstream target of HCG18. HCG18 promotes the proliferation, migration, and growth of ccRCC by inhibiting the activity of miR-152-3p (92). Moreover, RAB14 was identified as a downstream target of miR-152-3p; overexpression of RAB14 mitigated the effects of HCG18 knockdown on cell viability and metastasis. This indicates that HCG18 positively regulates the expression of RAB14 by sponging miR-152-3p, thereby promoting the progression of ccRCC and highlighting the inhibitory effect of miR-152-3p on ccRCC (92). Additionally, Jasinski-Bergner et al. (93) demonstrated that miR-152 exhibits a strong affinity for the 3′-UTR of human leukocyte antigen G (HLA-G), a gene commonly overexpressed in RCC, thereby shielding tumor cells from immune cell-mediated cytotoxicity (94). This implies that miR-152 can suppress HLA-G, enhancing immune cell-mediated killing of tumor cells and impeding RCC progression.

## The potential of miR-152 as a biomarker for urological malignancies

Biomarkers play a crucial role in providing valuable cancer-related information for the early screening, diagnosis, treatment, and prognosis of cancer. The traditional prostate cancer diagnostic indicator, blood PSA, has faced criticism in recent years due to its lack of specificity, false positives, and potential for overdiagnosis in benign prostate disorders (5), As PSA levels do not directly correlate with prostate cancer staging, there is a need for biomarkers with higher specificity and sensitivity in clinical management. Dysregulation of miRNA has been observed in various cancers (95, 96), with miRNA signatures proving to be more accurate than PSA in detecting different cancer types (97–99). The expression differences of miR-152 in prostate cancer compared to non-cancerous tissues, along with its correlation with Gleason score and pathological staging, suggest its potential as a diagnostic and prognostic biomarker for prostate cancer.

Liu et al. investigated the use of miR-146a and miR-152 in prostate cancer and their association with clinicopathological parameters. They found that the serum levels of miR-152 in prostate cancer patients were significantly lower compared to normal levels. The expression of miR-152 was strongly linked to clinical stage, presence of bone metastasis, and pathological stage. The diagnostic accuracy of prostate cancer using miR-152 was evaluated with an area under the curve (AUC) of 0.699 and a specificity of 94.64%. Furthermore, combined detection with miR-146a showed higher sensitivity than individual detection, suggesting that monitoring changes in the expression of miR-146a and miR-152 in serum could enhance the diagnostic precision of prostate cancer (100). Moya et al. also identified four significantly up-regulated miRNAs in the plasma of prostate cancer patients, including miR-152. These miRNAs exhibited high specificity and sensitivity in diagnosing prostate cancer. The AUC suggested that their diagnostic capability exceeded that of PSA, indicating that miRNA-152 in plasma holds promise as a diagnostic biomarker for prostate cancer (6, 101). Circulating miR-152 has shown promise in early detection of postoperative recurrence of prostate cancer. Chen et al. found that miR-152 levels in the serum of prostate cancer patients were significantly lower compared to controls, with even lower levels in patients experiencing postoperative recurrence. The ROC curve analysis demonstrated an AUC of 0.906, sensitivity of 91.4%, and specificity of 80.6%, indicating the potential of miR-152 as a biomarker for early prediction of postoperative recurrence (102). A MATE analysis also highlighted 14 down-regulated miRNAs, including miR-152, with diagnostic and predictive potential in distinguishing prostate cancer from benign prostatic hyperplasia (103). For metastasis detection, Lin et al. identified miR-152 as a potential marker through bioinformatics modelling, further confirming its role in prostate cancer metastasis (104). Additionally, Lichner et al. discovered 25 differentially expressed miRNAs associated with biochemical failure risk post-prostatectomy, with miR-152 interacting with ERBB3 and influencing cell proliferation to predict biochemical failure risk. These findings suggest the utility of miR-152 in predicting postoperative recurrence and metastatic features of prostate cancer, as well as in guiding postoperative adjuvant therapy decisions (105).

Early diagnosis of bladder cancer is crucial for reducing mortality rates. While urine cytology is a simple and non-invasive method, its sensitivity is limited. Cystoscopy-guided biopsy is the gold standard but is invasive and inconvenient for cancer screening. Therefore, the search for more sensitive and non-invasive biomarkers continues. Serum miRNA has emerged as a promising option for bladder cancer diagnosis and prognosis. A study on genome-wide miRNA analysis identified six miRNAs, including miR-152, in serum with high accuracy and sensitivity levels, outperforming urinary cytology. Notably, miR-152 was linked to NMIBC tumor recurrence, indicating its clinical significance (106). Additionally, miR-152’s tumor suppressor role was hindered by DNA hypermethylation, suggesting its potential as an epigenetic biomarker for bladder cancer. Köhler et al. found that miR-152 had low expression and hypermethylation in bladder cancer cell lines, proposing its use as an epigenetic biomarker for the disease (107).

In a bioinformatics analysis of potential therapeutic targets for hypertension-associated renal cell carcinoma (RCC), miR-152-3p was identified as significantly associated with hypertension-associated RCC (108). Furthermore, five miRNAs, including miR-152-3p, were found to be dysregulated in the urine of RCC patients both before and after surgery. These miRNAs exhibited a strong correlation, indicating their potential utility as post-operative disease status markers (109).

In summary, miR-152 in serum demonstrates high specificity and sensitivity in diagnosing prostate cancer, aiding in early detection. It is associated with the pathological stage and Gleason score of prostate cancer. Furthermore, miR-152 facilitates early monitoring of postoperative prostate cancer recurrence and metastasis risk, complementing the limitations of PSA. This suggests that miR-152 holds significant promise as a biomarker for the diagnosis, treatment, and prognosis of prostate cancer. In bladder cancer, miR-152, alongside other miRNAs, shows high sensitivity across various stages and offers clinical value in predicting postoperative recurrence. Studies also link miR-152 to renal cell carcinoma, indicating its potential as a biomarker for postoperative follow-up, although further research is necessary to fully understand its clinical significance.

## Therapeutic strategies for tumours based on miRNA therapy

miRNAs play a significant role in cancer regulation and can be targeted for therapeutic purposes. Cancer-associated miRNAs, categorized as oncogenic or tumor-suppressor miRNAs, can be targeted through two mechanisms. For oncogenic miRNAs, miRNA inhibitors can be administered to patients to reduce or eliminate their activity by isolating and binding to the miRNAs (35). These inhibitors typically consist of reverse sequences that complement the miRNAs. Additionally, ceRNAs, such as lncRNAs, circRNAs, and pseudogene transcripts, act as natural miRNA inhibitors by binding to miRNAs and competing for their limited availability, thereby reducing their function. On the other hand, for tumor-suppressor miRNAs, miRNA mimics can be delivered to patients to replace the downregulated miRNAs (110). miRNA therapy for cancer may involve the use of miRNA mimics or inhibitors alone or in combination with chemotherapy, radiotherapy, and immunotherapy to enhance treatment efficacy (111).

Gene therapy, which involves the therapeutic delivery of nucleic acids into cancer cells, has been regarded as a promising approach for decades. Recently, the U.S. Food and Drug Administration (FDA) has approved several new therapies utilizing small interfering RNA (siRNA), signaling the onset of a new era in targeted therapy. In 2018, the FDA approved Patisiran, the first-ever siRNA-based drug, for the treatment of transthyretin-mediated amyloidosis, representing a significant milestone in the history of RNA interference (RNAi) technology (112). The following year, the FDA approved Givlaari, the second siRNA drug, which targets aminolevulinic acid synthase 1 (ALAS1) mRNA for the treatment of acute hepatic porphyria (113). In 2020, Lumasiran was approved by the FDA as a treatment for primary hyperoxaluria type 1 (PH1) (114). Furthermore, several studies investigating miRNA-based therapies for advanced cancers have progressed to phase II clinical trials, including TargomiR (a miR-16 mimic-based therapy) for mesothelioma (115), Cobomarsen (an anti-miR-155 therapy) (116, 117), and Miravirsen (an anti-miR-122 therapy) for individuals infected with hepatitis C (118). The number of siRNA-based drugs is anticipated to increase significantly in the future, as siRNA-based therapies emerge as a promising treatment option that could revolutionize the clinical management of numerous diseases due to their capacity to selectively modulate protein expression. Nevertheless, siRNA-based cancer treatments must still address several challenges. One major challenge is identifying the most suitable miRNA candidate or target for each type of cancer (119). Since a single miRNA can have multiple targets, including both oncogenes and tumor suppressor genes, miRNAs may have dual roles in cancer progression, either promoting tumor development or inhibiting tumor progression, depending on various factors such as the cumulative effect of all targets and the specific tumor microenvironment. Using miRNAs as therapeutic targets requires a delicate balance between their oncogenic and tumor-suppressing effects to ensure their clinical efficacy. Another challenge is the lack of tissue-specific targeting of miRNAs delivered to the body, which may lead to unintended uptake by other tissues and subsequent adverse reactions. To address this issue, researchers have developed vectors, such as viral particles and nanoparticles, to facilitate targeted delivery of miRNAs to specific tissues. Additionally, the degradation of oligonucleotides by serum and intracellular RNA enzymes upon entry into the body poses a problem. To mitigate this, scientists have modified the chemical properties of oligonucleotides by adding phosphorothioate groups to the nucleotide or RNA backbone, thereby preventing degradation. These delivery vectors not only aid in targeting but also provide protection by encapsulating the miRNA. Furthermore, the potential toxicity, immune response, and off-target effects of miRNAs and vectors post-administration into the body are important considerations that must be addressed.

Despite the existing challenges associated with miRNA therapy for cancer treatment, addressing these issues could lead to significant breakthroughs in the management of various diseases, including cancer. Notably, miR-152 has demonstrated considerable promise in the treatment of cancer, particularly in relation to urinary malignancies. Studies indicate that miR-152 functions as a tumor-suppressor miRNA with decreased expression in prostate, bladder, and renal cell carcinomas. *In vitro* experiments have shown that overexpression of miR-152 can inhibit cancer cell proliferation, migration, invasion, angiogenesis, and promote apoptosis. This tumor-suppressor effect has also been validated *in vivo* through tumor xenografts (76, 81). Consequently, miR-152 emerges as a strong candidate for miRNA-based therapy in urological malignancies, with *in vivo* delivery of miR-152 mimics presenting a viable therapeutic approach. Ongoing advancements in miRNA carriers and chemical modifications hold promise for addressing targeting and stability issues associated with miR-152 treatment. However, further preclinical studies are necessary to assess the safety and efficacy of this approach.

## Conclusion and prospects

This review provides a comprehensive summary of the relationship between miR-152 and urological tumors (**Table 1**). Current research indicates that miR-152 acts as a tumor-suppressor miRNA in prostate, bladder, and renal cell carcinomas. By targeting downstream genes and signaling pathways associated with the biological behaviors of cancer cells, miR-152 inhibits tumor progression. Furthermore, studies demonstrate that miR-152 interacts with multiple genes and signaling cascades, forming a protein-protein interaction network that plays a role in the pathophysiological process of urological malignancies. These findings suggest that miR-152 could be a promising gene target for the treatment of urological malignancies. In the realm of urological malignancies, the expression of miR-152 exhibits notable variations and is closely linked to key pathological parameters such as cancer stage, grading, and metastasis. This suggests that miR-152 holds promise as a biomarker for the detection, treatment, and prognosis of urological malignancies. For instance, in prostate cancer, serum miR-152 demonstrates high sensitivity and specificity in diagnosing the disease, as well as in monitoring metastasis and postoperative recurrence. Similarly, in bladder cancer, serum miR-152 exhibits high sensitivity across different stages of the disease, surpassing the sensitivity of urocytology. This indicates that miR-152 could serve as a non-invasive biomarker with significant potential in the realm of urological malignancies. However, there are certain limitations in the current research on miR-152 in urological malignancies. Firstly, miR-152 remains relatively understudied in the urinary system, particularly in renal cell carcinoma, and the precise mechanism of miR-152 in urological malignancies remains poorly understood. Secondly, there is a lack of information on the stability of miR-152 and its targeting of tissues *in vivo* in current studies on miR-152 and urinary malignancies. Lastly, existing studies are predominantly focused on cellular and animal models, with a scarcity of clinical research. Numerous challenges need to be addressed before miR-152 can be effectively utilized in clinical settings. Once these challenges are overcome, miR-152 has the potential to emerge as an innovative therapeutic avenue for urological malignancies.

**Table 1:** miR-152 and its targets in urological malignancies.

Tumour type	Expression	Upstream Targets	Downstream Targets	References
Prostate cancer	Down	NA	KLF4	(79)
	Down	circANKS1B	TGFα	(69, 74)
	Down	NA	TMEM97	(74)
	Down	HOTAIR	FOXR2	(75)
	Down	LncRNA SNHG3	SLC7A11	(81)
	Down	NA	HMGA2	(90)
Bladder cancer	Down	NA	DNMT1	(60)
	Down	NA	DNMT1	(65)
	NA	lncRNA CCAT1	NA	(91)
	Down	NA	KIF14	(89)
Renal cell carcinoma	Down	LncRNA HCG18	RAB14	(92)
	NA	NA	HLA-G	(93)

In conclusion, miR-152 has demonstrated significant promise as both a therapeutic target and biomarker for urological malignancies. However, there are unresolved issues that must be addressed before miR-152 can realize its full potential in the treatment of urological malignancies and other types of tumors. Further research is essential to expand our understanding in this field.
